# Nanocomposite Biomaterials for Tissue-Engineered Hernia Repair: A Review of Recent Advances

**DOI:** 10.3390/biom15091348

**Published:** 2025-09-22

**Authors:** Octavian Andronic, Alexandru Cosmin Palcau, Alexandra Bolocan, Alexandru Dinulescu, Daniel Ion, Dan Nicolae Paduraru

**Affiliations:** 1 General Surgery Department, Carol Davila University of Medicine and Pharmacy, 050474 Bucharest, Romania; octavian.andronic@umfcd.ro (O.A.); alexandra.bolocan@umfcd.ro (A.B.); alexandru.dinulescu@drd.umfcd.ro (A.D.); daniel.ion@umfcd.ro (D.I.); dan.paduraru@umfcd.ro (D.N.P.); 2 Innovation and eHealth Center, Carol Davila University of Medicine and Pharmacy, 050474 Bucharest, Romania; 3 IIIrd Clinic of General and Emergency Surgery, University Emergency Hospital of Bucharest, 050098 Bucharest, Romania; 4 Department of Pediatrics, “Grigore Alexandrescu” Emergency Hospital for Children, 011743 Bucharest, Romania

**Keywords:** hernia repair, tissue engineering, nanocomposites, nanomedicine, regenerative medicine

## Abstract

Hernia repair is among the most frequent procedures in general surgery, traditionally performed with synthetic meshes such as polypropylene. While effective in reducing recurrence, these materials are biologically inert and often trigger chronic inflammation, fibrosis, pain, and impaired abdominal wall function, with a significant impact on long-term quality of life. A comprehensive literature search was conducted in PubMed, Web of Science, and Scopus databases, and relevant preclinical, clinical, and review articles were synthesized within a narrative review framework. Recent advances in tissue engineering propose a shift from passive reinforcement to regenerative strategies based on biomimetic scaffolds, nanomaterials, and nanocomposites that replicate the extracellular matrix, enhance cell integration, and provide controlled drug delivery. Nanotechnology enables localized release of anti-inflammatory, antimicrobial, and pro-angiogenic agents, while electrospun nanofibers and composite scaffolds improve strength and elasticity. In parallel, 3D printing allows for patient-specific implants with tailored architecture and regenerative potential. Although preclinical studies show encouraging results, clinical translation remains limited by cost, regulatory constraints, and long-term safety uncertainties. Overall, these innovations highlight a transition toward personalized and regenerative hernia repair, aiming to improve durability, function, and patient quality of life.

## 1. Introduction

The surgical repair of abdominal wall hernias represents a cornerstone of modern general surgery, with millions of procedures performed globally each year. For decades, the conventional approach has relied on the implantation of synthetic polymer meshes, primarily those made from polypropylene [1]. These meshes function as a static mechanical buttress, providing structural support to the weakened abdominal wall. While this technique has significantly reduced the immediate risk of hernia recurrence compared to older, tension-based suture repairs, it is not without a significant set of clinical and patient-related challenges [2]. A substantial number of patients report chronic postoperative pain, persistent foreign body sensation, and discomfort, all of which are directly linked to the mesh’s physical properties and its inability to fully integrate with the host’s biological environment [3,4]. The non-degradable nature of these materials often triggers a chronic inflammatory response, leading to dense scar tissue formation and a rigid, unphysiological abdominal wall. This reality underscores the critical need for a new generation of materials and a fundamentally different surgical philosophy [5,6].

An increasingly recognized dimension in the evaluation of outcomes after hernia repair is patient quality of life (QoL). While traditional metrics such as recurrence rates, complication rates, and short-term morbidity have long dominated surgical assessment, these parameters often fail to capture the broader impact of surgery on daily functioning and overall well-being. Chronic postoperative pain, restricted mobility, impaired abdominal wall biomechanics, and the persistent foreign body sensation associated with synthetic meshes can significantly diminish QoL, even in technically “successful” repairs. Patient-reported outcome measures (PROMs) have therefore emerged as essential tools, providing a comprehensive evaluation of physical, psychological, and social domains affected by hernia surgery. By integrating QoL into routine follow-up, clinicians and researchers can better appreciate the true burden of mesh-related morbidity and more accurately gauge the benefits of innovative, tissue-engineered approaches designed to restore not only structural integrity but also long-term functional and life-quality outcomes.

This review delves into the paradigm shift from traditional, passive reinforcement to an active, regenerative strategy founded on tissue engineering. This innovative field is transforming the landscape of hernia repair by focusing on the development of scaffolds that not only provide initial mechanical stability but also actively guide and promote the regeneration of native, functional abdominal wall tissue. The central focus of this review is on biomimetic materials—scaffolds engineered to closely mimic the complex biochemical and biomechanical architecture of the natural extracellular matrix (ECM) [7]. By replicating the nanoscale features and molecular cues of the ECM, these materials are designed to facilitate cell adhesion, proliferation, and differentiation, thereby fostering a more natural and physiologically sound repair [8,9].

Furthermore, we will explore how advancements in nanomedicine and nanotechnology are enhancing the regenerative capabilities of these biomimetic scaffolds. The unique properties of nanomaterials—including their high surface area-to-volume ratio and ability to be functionalized at the molecular level—are leveraged to create smarter implants [10,11]. We will discuss the incorporation of nanoparticles for a range of therapeutic purposes, from delivering growth factors to preventing infection, and the use of nanocomposites to fine-tune the mechanical properties of scaffolds. By integrating a reinforcing nanofiller (such as carbon nanotubes or graphene) into a polymeric matrix, these nanocomposites can achieve a superior balance of strength and elasticity, more closely mirroring the native biomechanics of the abdominal wall. The culmination of these efforts is the development of advanced scaffolds that not only prevent recurrence but also lead to true tissue regeneration, resulting in an outcome that is durable, biologically compatible, and free from the long-term complications associated with conventional methods [12]. This review differs from previous publications by specifically addressing the convergence of nanotechnology and tissue engineering in the context of hernia repair. Unlike earlier reviews that primarily focus on conventional meshes or on general biomaterials, our manuscript highlights the unique advantages of nanocomposite scaffolds, such as their ability to mimic the extracellular matrix, improve cellular interactions, and reduce long-term complications.

## 2. Material and Methods

This work was conducted as a narrative review aiming to summarize recent advances in tissue engineering and nanocomposite biomaterials for hernia repair. A comprehensive literature search was performed in PubMed, Web of Science, and Scopus databases. The search strategy combined keywords related to hernia repair, tissue engineering, nanotechnology, nanocomposites, 3D printing and biomaterials. Articles published in English between 2010 and 2025 were considered.

Both preclinical (in vitro and in vivo experimental studies) and clinical studies were included, along with relevant systematic reviews and meta-analyses. Conference abstracts, case reports, and publications not directly addressing abdominal wall hernia repair were excluded. References from selected articles were also screened manually to identify additional relevant publications. Given the narrative nature of this review, no formal quality assessment or meta-analysis was performed; instead, emphasis was placed on synthesizing conceptual advances, technological innovations, and translational challenges reported in the literature.

## 3. Results and Discussion

### 3.1. Hystorical Perspective

The evolution of biomaterials in hernia repair reflects a progressive understanding of tissue-implant interactions and the biomechanical requirements for successful surgical outcomes. Early hernia repair techniques relied primarily on autologous tissues and simple suturing methods, which were associated with high recurrence rates due to excessive tension on native tissues. The introduction of synthetic meshes in the mid-20th century, particularly polypropylene and polyester materials, represented a paradigmatic shift toward tension-free repair techniques that significantly reduced recurrence rates. However, clinical experience revealed complications associated with permanent synthetic implants, including chronic pain, mesh shrinkage, and foreign body reactions, prompting the development of absorbable materials such as polyglycolic acid and polylactic acid meshes. Contemporary research has focused on bioengineered solutions, including acellular dermal matrices, composite meshes with varying degradation profiles, and tissue-engineered constructs that promote native tissue regeneration while providing temporary mechanical support. This historical trajectory demonstrates the ongoing challenge of balancing mechanical strength, biocompatibility, and long-term integration in hernia repair biomaterials, with current investigations emphasizing patient-specific approaches and the optimization of host-material interactions [7,13].

### 3.2. Current State of Hernia Repair and Its Limitations

Today, the standard of care for abdominal wall hernia repair is the use of synthetic meshes. These materials, primarily made from non-degradable polymers such as polypropylene (PP) and expanded polytetrafluoroethylene (ePTFE), have been instrumental in reducing hernia recurrence rates by providing a passive, durable mechanical reinforcement. The Lichtenstein and tension-free hernia repair techniques, which rely on these meshes, have become the most widely accepted surgical approaches globally [14].

Although synthetic meshes are considered biologically inert, their implantation consistently triggers an inflammatory response. Once introduced into the host, they are initiating a cascade of chronic inflammation that ultimately results in fibrotic encapsulation. This process generates dense and rigid scar tissue around the implant. While such fibrosis provides structural reinforcement, it simultaneously gives rise to clinically significant drawbacks, including chronic postoperative pain, persistent foreign body sensation, and reduced abdominal wall compliance and elasticity [2,4]. Furthermore, the lack of biological integration can result in mesh migration, infection, and seroma formation, all of which compromise the long-term success of the repair [15]. These persistent issues highlight a fundamental mismatch between the biomechanical demands of the abdominal wall and the static, non-integrated nature of traditional synthetic meshes, creating a clear clinical imperative for a more biologically compatible and regenerative solution [16].

### 3.3. The Foundational Role of Tissue Engineering in Hernia Repair

To overcome the inherent limitations of static synthetic meshes, the field of tissue engineering offers a paradigm-shifting approach. This interdisciplinary field seeks to develop biological substitutes that can restore, maintain, or improve tissue function. In the context of hernia repair, tissue engineering moves beyond simple mechanical reinforcement towards a strategy of genuine tissue regeneration. The core principle involves a synergistic combination of three key components: a biomaterial scaffold, appropriate cells, and specific biochemical signals [17,18].

The scaffold is the foundational element, acting as a temporary, three-dimensional template that guides and facilitates the growth of new tissue. An ideal scaffold for hernia repair must meet several critical criteria: it must possess adequate mechanical strength to withstand intra-abdominal pressures, exhibit excellent biocompatibility to minimize immune response, and be biodegradable at a rate that allows for a gradual load transfer to the newly formed, native tissue. Unlike traditional meshes that are meant to be permanent, these scaffolds are designed to gradually degrade as the host’s own cells populate the structure, synthesize a new extracellular matrix (ECM), and effectively replace the scaffold with functional, neotissue [19,20].

The second component involves the interaction with cells, either sourced from the patient (autologous) or recruited from the surrounding host tissue. The scaffold’s architecture and surface chemistry are engineered to promote cell adhesion, proliferation, and differentiation. This cell seeding process is vital for initiating the regeneration cascade, as the cells begin to secrete their own ECM, ultimately rebuilding the abdominal wall [21].

Finally, biochemical signals, such as growth factors and cytokines, are often incorporated into the scaffold to orchestrate the regenerative process. These signals act as molecular cues, directing cell behavior and promoting tissue remodeling, angiogenesis (the formation of new blood vessels), and proper innervation [22]. By integrating these three components, tissue engineering provides a framework for creating a bio-integrated repair that is not just strong, but also physiologically resilient and adaptive to the patient’s body.

### 3.4. Biomimetic Materials: Mimicking the Extracellular Matrix

The primary objective of modern tissue-engineered hernia repair is to create scaffolds that are not simply biocompatible, but truly biomimetic—that is, they are designed to mimic the structural, biochemical, and biomechanical properties of the native extracellular matrix (ECM). The ECM provides not only mechanical support but also crucial biochemical and physical cues that regulate cell behavior, including adhesion, migration, and differentiation. By replicating this natural microenvironment, biomimetic scaffolds aim to guide a more natural and physiologically sound healing process, minimizing the inflammatory response and encouraging true tissue integration [23,24].

Materials for these scaffolds are often derived from natural sources, such as collagen, elastin, silk, or fibrin. Collagen and elastin, key components of the native abdominal wall, are particularly promising due to their inherent biocompatibility and ability to support cell growth [25,26]. For instance, scaffolds made from collagen can be engineered to possess a porous structure that facilitates cell infiltration and vascularization, while their inherent signals promote fibroblast proliferation and new collagen synthesis by the host [27]. Similarly, silk fibroin scaffolds are highly valued for their exceptional mechanical strength, slow degradation rate, and low immunogenicity, making them an excellent candidate for providing long-term support during the tissue remodeling phase [28,29].

A critical aspect of this biomimetic approach is the restoration of the abdominal wall’s natural biomechanics. The abdominal wall is a dynamic structure, constantly subjected to complex tensile, compressive, and torsional forces during everyday activities such as breathing, coughing, and movement [30]. Unlike traditional meshes, which are often stiff and isotropic (behaving the same in all directions), biomimetic scaffolds are engineered to align with the body’s natural lines of tension and torsion. This can be achieved through specific fiber alignment techniques or by incorporating materials with anisotropic properties. By matching the mechanical compliance and flexibility of the native tissue, these advanced scaffolds prevent the formation of a rigid repair, significantly reducing long-term complications such as chronic pain and discomfort and ensuring that the newly formed tissue can withstand physiological stresses without deforming or failing [31,32].

### 3.5. Nanomaterials and Nanocomposites: Enhancing Hernia Repair at the Nanoscale

The evolution of tissue engineering for hernia repair is inextricably linked to the advancements in nanomedicine. Working at the nanoscale (1–100 nm) allows for a level of control that directly influences cellular behavior, as cells and the native extracellular matrix (ECM) operate at this same dimensional scale. This nanoscopic control enables the creation of “smart” scaffolds with enhanced biological and mechanical properties, far superior to those of traditional materials [33].

A key application in nanocomposite hernia repair biomaterials is the strategic use of nanoparticles as functional additives, which can be precisely loaded with specific therapeutic agents and seamlessly integrated within the three-dimensional scaffold architecture. These nanocarriers provide unprecedented control over drug release kinetics, enabling sustained, localized delivery of bioactive molecules at therapeutically relevant concentrations while minimizing systemic exposure and associated side effects—a critical advancement for orchestrating the complex, multi-phase healing process [34]. The controlled release mechanism can be fine-tuned through various parameters including nanoparticle composition, size distribution, surface modifications, and encapsulation techniques, allowing for tailored release profiles that match the temporal requirements of different healing phases.

To address the inevitable inflammatory response that occurs post-implantation—a natural but potentially detrimental biological reaction—scaffolds can be functionalized with biodegradable nanoparticles loaded with potent anti-inflammatory agents such as dexamethasone, prednisolone, or newer selective anti-inflammatory compounds [35,36]. This targeted, spatiotemporally controlled release helps modulate the early inflammatory cascade by regulating key inflammatory mediators such as tumor necrosis factor-alpha (TNF-α), interleukin-1β (IL-1β), and nuclear factor-κB (NF-κB) signaling pathways. By maintaining anti-inflammatory drug concentrations within the therapeutic window for extended periods, this approach effectively prevents the transition from acute to chronic inflammation that typically leads to excessive scar tissue formation, fibrotic encapsulation, mesh shrinkage, and chronic pain syndromes that can significantly impact patient quality of life.

Similarly, to actively promote new tissue growth, vascularization, and functional tissue integration, nanoparticles can be loaded with specific growth factors such as VEGF (Vascular Endothelial Growth Factor), FGF (Fibroblast Growth Factor), PDGF (Platelet-Derived Growth Factor), or TGF-β (Transforming Growth Factor-beta), each targeting different aspects of the regenerative process [37,38,39]. The controlled, gradual release of these bioactive proteins stimulates angiogenesis through the formation of new capillary networks, recruits endogenous host cells including fibroblasts, mesenchymal stem cells, and endothelial progenitor cells to the repair site, and encourages these cells to synthesize a new, mechanically competent extracellular matrix (ECM) that closely resembles native abdominal wall tissue in both composition and mechanical properties.

To prevent surgical site infections—one of the most severe and costly complications in hernia repair surgery—broad-spectrum antibiotics such as vancomycin, gentamicin, rifampin, or newer antimicrobial peptides can be encapsulated within biodegradable nanoparticles and homogeneously distributed throughout the scaffold matrix [40,41]. This innovative drug delivery approach provides sustained, localized antimicrobial protection for weeks to months post-implantation, maintaining therapeutic concentrations at the implant-tissue interface while significantly reducing the risk of both early and late-onset infections. Importantly, this localized delivery strategy eliminates the need for high systemic antibiotic doses, thereby reducing the risk of systemic toxicity, antibiotic-associated complications, and the development of antibiotic-resistant bacterial strains—a growing concern in modern surgical practice [40,41,42].

Furthermore, the scaffold itself can be fabricated at the nanoscale. The electrospinning process is a powerful technique used to create non-woven fibrous meshes with fiber diameters in the nanometer range [43,44]. This method involves using a high-voltage electric field to draw a charged polymer solution from a syringe into a solid fiber. The setup typically consists of a syringe pump containing the polymer solution, a high-voltage power supply, and a grounded collector plate [45]. As the electric potential difference between the syringe tip and the collector increases, the charged polymer solution forms a “Taylor cone” from which a thin jet is ejected. This jet rapidly elongates and thins as it travels through the air, and the solvent evaporates, leaving behind solid nanofibers that collect on the grounded plate, forming a highly porous, interconnected mat. The resulting nanofiber scaffolds closely mimic the morphology of the native ECM, with a high surface-area-to-volume ratio that promotes superior cell adhesion, proliferation, and infiltration compared to conventional macro-porous meshes. This structural biomimicry is critical for guiding a more organized and robust tissue ingrowth [46].

Finally, the most advanced approach involves the development of nanocomposites. These materials combine a polymer matrix with a nanofiller (e.g., carbon nanotubes, graphene oxide, or nanocrystalline hydroxyapatite). This synergy creates a composite material with significantly improved properties. For example, the addition of carbon nanotubes can dramatically increase the mechanical strength and elasticity of a polymer scaffold, allowing it to withstand the dynamic forces of the abdominal wall without becoming rigid. Nanocomposites represent the pinnacle of this approach, offering a unique blend of structural integrity and biological functionality that is essential for a truly regenerative and durable hernia repair [26,47,48].

### 3.6. 3D Printing and Personalized Scaffolds

An emerging and highly promising direction in abdominal wall reconstruction is the application of 3D printing technologies for the design and fabrication of biocompatible scaffolds. Unlike traditional meshes, which are manufactured in standardized forms, 3D printing allows for the creation of patient-specific implants, tailored precisely to the size, shape, and biomechanical characteristics of the hernia defect. This personalized approach reduces the risk of mechanical mismatch, improves anatomical integration, and potentially minimizes long-term complications such as pain, stiffness, or recurrence [49,50].

A major advantage of 3D printing lies in its ability to incorporate complex architectural features that mimic the hierarchical organization of the native extracellular matrix. Techniques such as fused deposition modeling (FDM), selective laser sintering (SLS), and extrusion-based bioprinting enable the fabrication of scaffolds with controlled porosity, anisotropic fiber alignment, and tunable degradation rates, all of which are essential for guiding cell infiltration, angiogenesis, and gradual tissue remodeling [51].

Selective Laser Sintering (SLS) uses a high-powered laser to selectively fuse powdered materials layer by layer according to digital cross-sectional data. The powder bed is preheated below the material’s melting point, allowing precise sintering of particles while unsintered powder provides structural support. This technique enables complex internal geometries and interconnected porous networks without requiring additional support structures, making it ideal for intricate scaffold architectures with controlled porosity [52,53,54].

Fused Deposition Modeling (FDM) extrudes heated thermoplastic filaments through a controlled nozzle, depositing material layer by layer to build three-dimensional structures. The filament is heated to its melting point and extruded through temperature-controlled nozzles, with each layer fusing to the previous one upon cooling. FDM offers versatility in biocompatible material selection, including PLA, PCL, and PLGA, and enables multi-material printing for constructs with varying mechanical properties [55,56,57].

Extrusion bioprinting represents a specialized technique designed for biological materials, using pneumatic or mechanical systems to extrude bioinks—hydrogel formulations containing cells and bioactive factors—through fine nozzles at physiological conditions. The bioink requires optimal rheological properties for smooth extrusion and rapid gelation to maintain shape fidelity while preserving cell viability. Advanced systems can simultaneously deposit multiple bioinks, enabling multi-layered constructs that combine structural polymers with cell-laden hydrogels for mechanically robust yet biologically active hernia repair materials [58,59,60].

Various biomaterials have been explored for 3D-printed scaffolds in hernia repair. Synthetic polymers such as polycaprolactone (PCL) and polylactic acid (PLA) provide favorable mechanical properties and can be combined with natural polymers (e.g., collagen, gelatin, silk fibroin) to enhance biocompatibility. Hybrid scaffolds, integrating biodegradable polymers with nanofillers like graphene oxide or hydroxyapatite, have also been investigated to improve elasticity and strength while maintaining cellular compatibility. In addition, bio-inks loaded with growth factors or stem cells are being developed to produce constructs with regenerative potential, moving beyond passive reinforcement toward active participation in tissue repair [10,61].

Early preclinical studies demonstrate encouraging outcomes. For example, 3D-printed collagen–PCL scaffolds implanted in animal models showed superior vascularization, reduced inflammatory response, and more natural integration compared to conventional meshes [62]. Furthermore, computational modeling combined with 3D printing enables the fabrication of patient-specific implants, designed based on preoperative imaging, representing a step toward personalized regenerative surgery.

While translation into routine clinical practice remains limited by regulatory, cost, and standardization challenges, the integration of 3D printing with tissue engineering represents a transformative pathway. By combining personalized design, biomimetic architecture, and the incorporation of bioactive cues, 3D-printed scaffolds have the potential to redefine the paradigm of hernia repair, shifting from static mechanical reinforcement to dynamic, regenerative, and patient-centered solutions [63].

### 3.7. Industrial Status and Translation of Nanocomposite Biomaterials

Although promising in preclinical and early clinical studies, the industrial translation of nanocomposite biomaterials for hernia repair remains in its infancy. A limited number of products have reached commercialization, and most remain in experimental or prototype stages. Challenges include scaling up reproducible manufacturing processes, ensuring batch-to-batch consistency, and meeting stringent regulatory requirements for safety and biocompatibility. Nevertheless, collaborations between academia, industry, and regulatory agencies are accelerating this process, and the next decade may witness the broader clinical adoption of these advanced materials [18,64].

### 3.8. Current Clinical Status and Regulatory Landscape

The translation of advanced nanocomposite biomaterials from laboratory to clinical practice requires careful consideration of the existing regulatory and commercial landscape. Currently, several biologic and biosynthetic meshes have received FDA approval and are in routine clinical use, providing valuable precedents for novel material approval pathways. FDA-approved biologic meshes include acellular dermal matrices such as AlloDerm^®^ (LifeCell Corporation, Branchburg, NJ, USA), FlexHD^®^ (Ethicon, Raritan, NJ, USA), and Strattice™ (Acelity, San Antonio, TX, USA), which have demonstrated clinical efficacy in complex hernia repairs while establishing safety profiles for tissue-derived materials. Biosynthetic options like Phasix™ (Becton Dickinson, Franklin Lakes, NJ, USA), a fully absorbable mesh composed of poly-4-hydroxybutyrate, represent successful examples of synthetic biodegradable materials achieving clinical adoption [65,66,67,68].

Regarding advanced manufacturing technologies, several clinical trials are currently investigating 3D-printed and nano-functionalized implants for hernia repair. Notable ongoing studies include Phase II trials of patient-specific 3D-printed meshes (ClinicalTrials.gov identifier: NCT04521205) and early-phase investigations of growth factor-loaded nanofiber meshes for complex abdominal wall reconstruction. The regulatory pathway for these advanced materials typically follows the FDA’s 510(k) premarket notification process for devices with predicate equivalents, or the more stringent Premarket Approval (PMA) pathway for novel technologies without established precedents. The European Medicines Agency (EMA) has similarly established guidelines for advanced therapy medicinal products (ATMPs) that encompass tissue-engineered constructs, providing a regulatory framework that encourages innovation while ensuring patient safety [63,69,70].

### 3.9. Challenges and Future Directions

Despite the remarkable progress in tissue-engineered hernia repair, the transition from promising laboratory results to widespread clinical application faces significant hurdles. A primary challenge lies in the regulatory approval process, which for novel biomaterials is often protracted and complex, requiring extensive preclinical and clinical data to demonstrate long-term safety and efficacy. Additionally, the scalability and cost-effectiveness of manufacturing these advanced scaffolds pose a practical barrier to their clinical adoption. The production of intricate, biomimetic, and nano-enhanced materials is considerably more expensive than traditional mass-produced synthetic meshes [16].

Another critical area that demands further research is the long-term performance of these regenerative scaffolds. While they show excellent potential for tissue integration and reduced inflammation in short-term studies, more extensive in vivo trials are needed to confirm their durability and to fully understand their degradation profile and potential for long-term complications. The translation from in vitro to in vivo results remains a complex and unpredictable step, as the intricate biological environment of the human body can react in unexpected ways.

Looking ahead, the future of tissue engineering in hernia repair will likely focus on the development of truly “smart” and personalized materials. This includes:

- Dynamically responsive scaffolds: Materials that can sense the mechanical environment and adapt their properties in real time, perhaps by stiffening under stress and softening during rest, to better match the natural function of the abdominal wall.
- Patient-specific implants: The use of advanced imaging and computational modeling to design and 3D-print scaffolds tailored to an individual’s specific defect size, shape, and biomechanical needs.
- Multifunctional hybrid systems: Integrating multiple technologies, such as a strong, biodegradable core for initial support, a biomimetic surface for cell adhesion, and a nano-loaded layer for targeted drug delivery [26,71].

These future directions aim not only to improve the durability of the repair but also to usher in an era of personalized, patient-centric medicine that minimizes long-term morbidity and maximizes quality of life.

To provide a clearer overview of the current strategies in hernia repair, the following table (Table 1) summarizes the key features and advantages of each approach, alongside their main limitations and challenges. This comparative outline highlights the practical benefits as well as the potential drawbacks associated with different techniques, facilitating a balanced understanding of their clinical applicability.

## 4. Conclusions

Conventional synthetic meshes, though effective in reducing recurrence, remain limited by chronic inflammation, fibrosis, pain, and poor biomechanical integration. Advances in tissue engineering have introduced biomimetic scaffolds and nanocomposites that more closely mimic the extracellular matrix, promoting cell adhesion, angiogenesis, and controlled tissue remodeling. Nanoparticles further enhance these scaffolds by enabling local delivery of anti-inflammatory, antimicrobial, and pro-regenerative agents. In addition, 3D printing enables the development of patient-specific implants with tailored architecture and mechanical properties, offering promising paths toward personalized hernia repair.

Despite encouraging preclinical results, translation into clinical practice is still constrained by regulatory, cost, and long-term safety challenges. Continued research is needed to validate durability and optimize large-scale application. Collectively, these innovations represent a shift from static mechanical reinforcement toward regenerative, patient-centered solutions with the potential to improve long-term outcomes and quality of life.

This manuscript aims to serve as a comprehensive resource for scientists working in the fields of biomaterials, nanotechnology, and regenerative medicine. By synthesizing recent advances, identifying translational challenges, and highlighting areas for future exploration, it provides a framework to guide both fundamental research and clinical innovation in hernia repair.

## Figures and Tables

**Table 1:** Summary of Advances in Tissue-Engineered and Nanocomposite Approaches for Hernia Repair.

Section/Approach	Key Features and Advantages	Limitations/Challenges
**Conventional Synthetic Meshes**	Durable, widely available; effective in reducing recurrence rates; standardized techniques (e.g., Lichtenstein)	Biologically inert but induce chronic inflammation; fibrosis and rigidity; chronic pain, foreign body sensation; poor biomechanical integration; risk of infection/migration
**Tissue Engineering Principles**	Combines scaffold, cells, and biochemical cues; promotes genuine tissue regeneration; scaffold biodegrades and is replaced by functional tissue	Complexity of integrating all components; scalability; regulatory challenges
**Biomimetic Materials**	Derived from collagen, elastin, silk, fibrin; mimic ECM architecture and biomechanics; support cell adhesion, proliferation, angiogenesis	Variability of natural materials; potential immune response; long-term durability not fully established
**Nanomaterials and Nanocomposites**	Nanoscale control of cell–material interactions; nanoparticles for drug delivery, anti-inflammation, angiogenesis, antimicrobial action; electrospun nanofibers mimic ECM; nanofillers (CNTs, graphene, hydroxyapatite) improve strength and elasticity	Manufacturing cost and complexity; long-term safety; translation from in vitro/in vivo to clinical use
**3D Printing and Personalized Scaffolds**	Patient-specific design tailored to defect geometry; controlled porosity and anisotropy; integration of polymers, natural biomaterials, bio-inks with growth factors/stem cells; potential for regenerative and personalized repair	Limited clinical translation; high cost; regulatory hurdles; need for standardization and quality assurance
**Future Directions**	Smart scaffolds responsive to mechanical stress; multifunctional hybrid systems; personalized implants via imaging and computational modeling	Require advanced validation, clinical trials, and regulatory approval; cost-effectiveness remains uncertain

## Data Availability

No new data were created or analyzed in this study. Data sharing is not applicable to this article.

## References

[1] Jensen K.K., Henriksen N.A., Jorgensen L.N., Hope W.W., Cobb W.S., Adrales G.L. (2017). Inguinal Hernia Epidemiol. BT—Textbook of Hernia.

[2] Sandø A., Rosen M.J., Heniford B.T., Bisgaard T. (2020). Long-term patient-reported outcomes and quality of the evidence in ventral hernia mesh repair: A systematic review. Hernia.

[3] Ahmed U., Rosenberg J., Baker J.J. (2025). Chronic pain and foreign body sensation based on mesh placement in primary ventral hernia repair: A systematic review highlighting the evidence gap and a call to action. Langenbeck’s Arch. Surg..

[4] van Veenendaal N., Poelman M.M., Heuvel B.v.D., Dwars B.J., Schreurs W.H., Stoot J.H.M.B., Bonjer H.J. (2021). Patient-reported outcomes after incisional hernia repair. Hernia.

[5] Heymann F., von Trotha K.-T., Preisinger C., Lynen-Jansen P., Roeth A.A., Geiger M., Geisler L.J., Frank A.K., Conze J., Luedde T. (2019). Polypropylene mesh implantation for hernia repair causes myeloid cell–driven persistent inflammation. J. Clin. Investig..

[6] Saiding Q., Chen Y., Wang J., Pereira C.L., Sarmento B., Cui W., Chen X. (2023). Abdominal wall hernia repair: From prosthetic meshes to smart materials. Mater. Today Bio.

[7] Najm A., Niculescu A.-G., Gaspar B.S., Grumezescu A.M., Beuran M. (2023). A Review of Abdominal Meshes for Hernia Repair—Current Status and Emerging Solutions. Materials.

[8] Sung T.-C., Wang T., Liu Q., Ling Q.-D., Subbiah S.K., Renuka R.R., Hsu S.-T., Umezawa A., Higuchi A. (2023). Cell-binding peptides on the material surface guide stem cell fate of adhesion, proliferation and differentiation. J. Mater. Chem. B.

[9] Gómez-Gil V., Pascual G., Bellón J.M. (2019). Biomaterial Implants in Abdominal Wall Hernia Repair: A Review on the Importance of the Peritoneal Interface. Processes.

[10] Deveci M.Z.Y., Enguven G., Ege H., Alakus I., Agturk G., Yontem F.D., Yilmaz S., Kirgiz O., Akcakavak G., Kazak F. (2024). Multifunctional hernia repair biopatch: Development, characterization, in vitro and in vivo evaluation. J. Drug Deliv. Sci. Technol..

[11] Rodríguez M., Gómez-Gil V., Pérez-Köhler B., Pascual G., Bellón J.M. (2021). Polymer Hernia Repair Materials: Adapting to Patient Needs and Surgical Techniques. Materials.

[12] Ellis R., Miller B.T. (2023). Mesh Selection in Abdominal Wall Reconstruction. Surg. Clin. N. Am..

[13] Cortes R.A., Miranda E., Lee H., Gertner M.E., Rosen M.J. (2008). Biomaterials and the Evolution of Hernia Repair I: The History of Bio-materials and the Permanent Meshes. Atlas of Abdominal Wall Reconstruction.

[14] Messias B.A., Nicastro R.G., Mocchetti E.R., Waisberg J., Roll S., Junior M.A.F.R. (2024). Lichtenstein technique for inguinal hernia repair: Ten recommendations to optimize surgical outcomes. Hernia.

[15] Jangjoo A., Kalantari M.E., Zandbaf T. (2020). Mesh migration following abdominal hernia repair: A case report, and literature review. Casp. J. Intern. Med..

[16] Antoniadi E., Ferreira N.M., Vaz M.F., Parente M., Ferraz M.P., Silva E. (2025). Innovative Strategies in Hernia Mesh Design: Materials, Mechanics, and Modeling. Materials.

[17] Afewerki S., Bassous N., Harb S.V., Corat M.A.F., Maharjan S., Ruiz-Esparza G.U., de Paula M.M.M., Webster T.J., Tim C.R., Viana B.C. (2021). Engineering multifunctional bactericidal nanofibers for abdominal hernia repair. Commun. Biol..

[18] Kalaba S., Gerhard E., Winder J.S., Pauli E.M., Haluck R.S., Yang J. (2016). Design strategies and applications of biomaterials and devices for Hernia repair. Bioact. Mater..

[19] Pérez-Köhler B., Benito-Martínez S., Gómez-Gil V., Rodríguez M., Pascual G., Bellón J.M. (2021). New Insights into the Application of 3D-Printing Technology in Hernia Repair. Materials.

[20] Pantermehl S., Emmert S., Foth A., Grabow N., Alkildani S., Bader R., Barbeck M., Jung O. (2021). 3D Printing for Soft Tissue Regeneration and Applications in Medicine. Biomedicines.

[21] Krishani M., Shin W.Y., Suhaimi H., Sambudi N.S. (2023). Development of Scaffolds from Bio-Based Natural Materials for Tissue Regeneration Applications: A Review. Gels.

[22] Tyavambiza C., Meyer M., Meyer S. (2022). Cellular and Molecular Events of Wound Healing and the Potential of Silver Based Nanoformulations as Wound Healing Agents. Bioengineering.

[23] Bertsch C., Maréchal H., Gribova V., Lévy B., Debry C., Lavalle P., Fath L. (2023). Biomimetic Bilayered Scaffolds for Tissue Engineering: From Current Design Strategies to Medical Applications. Adv. Healthc. Mater..

[24] Liu S., Yu J.-M., Gan Y.-C., Qiu X.-Z., Gao Z.-C., Wang H., Chen S.-X., Xiong Y., Liu G.-H., Lin S.-E. (2023). Biomimetic natural biomaterials for tissue engineering and regenerative medicine: New biosynthesis methods, recent advances, and emerging applications. Mil. Med. Res..

[25] Costa A., Adamo S., Gossetti F., D’Amore L., Ceci F., Negro P., Bruzzone P., Amore D. (2019). Ceci Biological Scaffolds for Abdominal Wall Repair: Future in Clinical Application?. Materials.

[26] Heidari B.S., Dodda J.M., El-Khordagui L.K., Focarete M.L., Maroti P., Toth L., Pacilio S., El-Habashy S.E., Boateng J., Catanzano O. (2024). Emerging materials and technologies for advancing bioresorbable surgical meshes. Acta Biomater..

[27] He X., Li W., Liu S., Li Y., Chen Y., Dan N., Dan W., Zhu M. (2022). Fabrication of high-strength, flexible, porous collagen-based scaffolds to promote tissue regeneration. Mater. Today Bio.

[28] Radulescu D.-M., Andronescu E., Vasile O.R., Ficai A., Vasile B.S. (2024). Silk fibroin-based scaffolds for wound healing applications with metal oxide nanoparticles. J. Drug Deliv. Sci. Technol..

[29] Ma L., Dong W., Lai E., Wang J. (2024). Silk fibroin-based scaffolds for tissue engineering. Front. Bioeng. Biotechnol..

[30] Joppin V., Bendahan D., El Ahmadi A.A., Masson C., Bege T. (2025). Biomechanics of the abdominal wall before and after ventral hernia repair using dynamic MRI. Hernia.

[31] Minardi S., Taraballi F., Wang X., Cabrera F.J., Van Eps J.L., Robbins A.B., Sandri M., Moreno M.R., Weiner B.K., Tasciotti E. (2017). Biomimetic collagen/elastin meshes for ventral hernia repair in a rat model. Acta Biomater..

[32] Jing X., Hu X., Feng P., Liu Y., Yang J. (2022). Modification of nanofibrous scaffolds to mimic extracellular matrix in physical and chemical structuring. Polym. Eng. Sci..

[33] Farahani P.K. (2024). Nanotechnology approaches in abdominal wall reconstruction: A narrative review about scaffold and meshes. JPRAS Open.

[34] Yetisgin A.A., Cetinel S., Zuvin M., Kosar A., Kutlu O. (2020). Therapeutic Nanoparticles and Their Targeted Delivery Applications. Molecules.

[35] Wang H., Zhou Y., Sun Q., Zhou C., Hu S., Lenahan C., Xu W., Deng Y., Li G., Tao S. (2021). Update on Nanoparticle-Based Drug Delivery System for Anti-inflammatory Treatment. Front. Bioeng. Biotechnol..

[36] Xu D., Fang M., Wang Q., Qiao Y., Li Y., Wang L. (2021). Latest Trends on the Attenuation of Systemic Foreign Body Response and Infectious Complications of Synthetic Hernia Meshes. ACS Appl. Bio Mater..

[37] Weng T., Wang J., Yang M., Zhang W., Wu P., You C., Han C., Wang X. (2022). Nanomaterials for the delivery of bioactive factors to enhance angiogenesis of dermal substitutes during wound healing. Burn. Trauma.

[38] Chávez J.C.P., McGrath M., O’COnnor C., Dervan A., Dixon J.E., Kearney C.J., Browne S., O’BRien F.J. (2025). Development of a VEGF-activated scaffold with enhanced angiogenic and neurogenic properties for chronic wound healing applications. Biomater. Sci..

[39] Vijayan A., Nanditha C.K., Kumar G.S.V. (2021). ECM-mimicking nanofibrous scaffold enriched with dual growth factor carrying nanoparticles for diabetic wound healing. Nanoscale Adv..

[40] Pisani S., Tufail S., Rosalia M., Dorati R., Genta I., Chiesa E., Conti B. (2024). Antibiotic-Loaded Nano-Sized Delivery Systems: An Insight into Gentamicin and Vancomycin. J. Funct. Biomater..

[41] Mirel S., Pusta A., Moldovan M., Moldovan S. (2022). Antimicrobial Meshes for Hernia Repair: Current Progress and Perspectives. J. Clin. Med..

[42] Guillaume O., Pérez-Tanoira R., Fortelny R., Redl H., Moriarty T.F., Richards R.G., Eglin D., Puchner A.P. (2018). Infections associated with mesh repairs of abdominal wall hernias: Are antimicrobial biomaterials the longed-for solution?. Biomaterials.

[43] Li H., Yang W. (2016). Electrospinning technology in non-woven fabric manufacturing. Non-Woven Fabrics.

[44] Xiang C., Frey M.W. (2016). Increasing Mechanical Properties of 2-D-Structured Electrospun Nylon 6 Non-Woven Fiber Mats. Materials.

[45] Jalalah M., Ahmad A., Saleem A., Qadir M.B., Khaliq Z., Khan M.Q., Nazir A., Faisal M., Alsaiari M., Irfan M. (2022). Electrospun Nanofiber/Textile Supported Composite Membranes with Improved Mechanical Performance for Biomedical Applications. Membranes.

[46] Huang W., Xiao Y., Shi X. (2019). Construction of Electrospun Organic/Inorganic Hybrid Nanofibers for Drug Delivery and Tissue Engineering Applications. Adv. Fiber Mater..

[47] Houshyar S., Sarker A., Jadhav A., Kumar G.S., Bhattacharyya A., Nayak R., Shanks R.A., Saha T., Rifai A., Padhye R. (2020). Polypropylene-nanodiamond composite for hernia mesh. Mater. Sci. Eng. C.

[48] Mao Y., Meng Y., Li S., Li Y., Guidoin R., Qiao Y., Zhang Z., Brochu G., Tang J., Wang L. (2021). Comparative study on nanofiber containing polypropylene-based composite mesh for abdominal wall hernia repair. Mater. Des..

[49] Olmos-Juste R., Olza S., Gabilondo N., Eceiza A. (2022). Tailor-Made 3D Printed Meshes of Alginate-Waterborne Polyurethane as Suitable Implants for Hernia Repair. Macromol. Biosci..

[50] Wang F., Hou L., Shan Y.-H., Li Z.-S., Yang X.-F. (2024). Polyurethane-based three-dimensional printing for biological mesh carriers. Sci. Rep..

[51] Wang F., Yang X.-F. (2020). Application of computer tomography-based 3D reconstruction technique in hernia repair surgery. World J. Clin. Cases.

[52] Li T., Peng Z., Lv Q., Li L., Zhang C., Pang L., Zhang C., Li Y., Chen Y., Tang X. (2024). SLS 3D printing to fabricate poly(vinyl alcohol)/hydroxyapatite bioactive composite porous scaffolds and their bone defect repair property. ACS Biomater. Sci. Eng..

[53] Shirazi S.F., Gharehkhani S., Mehrali M., Yarmand H., Metselaar H.S., Adib Kadri N., Osman N.A. (2015). A review on powder-based additive manufacturing for tissue engineering: Selective laser sintering and inkjet 3D printing. Sci. Technol. Adv. Mater..

[54] Gibson I., Rosen D.W., Stucker B. (2015). Additive Manufacturing Technologies: Rapid Prototyping to Direct Digital Manufacturing.

[55] Ngo T.D., Kashani A., Imbalzano G., Nguyen K.T.Q., Hui D. (2018). Additive Manufacturing (3D Printing): A Review of Materials, Methods, Applications and Challenges. Compos. Part B Eng..

[56] Winarso R., Anggoro P.W., Ismail R., Jamari J., Bayuseno A.P. (2022). Application of fused deposition modeling (FDM) on bone scaffold manufacturing process: A review. Heliyon.

[57] Cano-Vicent A., Tambuwala M.M., Hassan S.S., Barh D., Aljabali A.A.A., Birkett M., Arjunan A., Serrano-Aroca Á. (2021). Fused deposition modelling: Current status, methodology, applications and future prospects. Addit. Manuf..

[58] Derakhshanfar S., Mbeleck R., Xu K., Zhang X., Zhong W., Xing M. (2017). 3D bioprinting for biomedical devices and tissue engineering: A review of recent trends and advances. Bioact. Mater..

[59] Paxton N., Smolan W., Böck T., Melchels F., Groll J., Jungst T. (2017). Proposal to assess printability of bioinks for extrusion-based bioprinting and evaluation of rheological properties governing bioprintability. Biofabrication.

[60] Chan J.P., Battiston K.G., Santerre J.P. (2019). Synthesis and characterization of electrospun nanofibrous tissue engineering scaffolds generated from in situ polymerization of ionomeric polyurethane composites. Acta Biomater..

[61] Hu Q., Zhang R., Zhang H., Yang D., Liu S., Song Z., Gu Y., Ramalingam M. (2021). Topological Structure Design and Fabrication of Biocompatible PLA/TPU/ADM Mesh with Appropriate Elasticity for Hernia Repair. Macromol. Biosci..

[62] Qamar N., Abbas N., Irfan M., Hussain A., Arshad M.S., Latif S., Mehmood F., Ghori M.U. (2019). Personalized 3D printed ciprofloxacin impregnated meshes for the management of hernia. J. Drug Deliv. Sci. Technol..

[63] Yadav P., Mukherjee A., Rajput J.H., Choudhari A.P., Poundarik A., Das B. (2025). Gelatin Multiwalled Carbon Nanotube Composite 3D Printed Semi Biological Mesh for Abdominal Hernia Treatment. Chem.–Asian J..

[64] Liu W., Xie Y., Zheng Y., He W., Qiao K., Meng H. (2020). Regulatory science for hernia mesh: Current status and future perspectives. Bioact. Mater..

[65] Huntington C.R., Cox T.C., Blair L.J., Schell S., Randolph D., Prasad T., Lincourt A., Heniford B.T., Augenstein V.A. (2016). Biologic mesh in ventral hernia repair: Outcomes, recurrence, and charge analysis. Am. J. Surg..

[66] Alzahrani A., Alhindi N., Alotaibi S., Alzibali K., Alaqla A.A., Alzahrani S., Alsallat I.M., Ghunaim M., Alharthi M. (2024). A systematic review and meta-analysis of randomized controlled trials for the management of ventral hernia: Biologic versus synthetic mesh. Updates Surg..

[67] Levy A.S., Bernstein J.L., Premaratne I.D., Rohde C.H., Otterburn D.M., Morrison K.A., Lieberman M., Pomp A., Spector J.A. (2021). Poly-4-hydroxybutyrate (Phasix™) mesh onlay in complex abdominal wall repair. Surg. Endosc..

[68] Tran D.H., Rubarth C., Leeds S.G., Fair L., McGowan T., Ramakrishnan S., Shabbir R., Ogola G., Ward M.A., Aladegbami B. (2024). The use of poly-4-hydroxybutyrate (P4HB, Phasix™) mesh in ventral hernia repair: A systematic review and meta-analysis. Hernia.

[69] ClinicalTrials.gov Study of Phasix™ Mesh for VHWG Grade 3 Midline Hernia. NCTA. https://clinicaltrials.gov/study/NCT02720042.

[70] International Hernia Mesh Registry. NCT. https://clinicaltrials.gov/study/NCT00622583.

[71] Goldenberg D., McLaughlin C., Koduru S.V., Ravnic D.J. (2021). Regenerative Engineering: Current Applications and Future Perspectives. Front. Surg..

